# A New Broadband and Strong Absorption Performance FeCO_3_/RGO Microwave Absorption Nanocomposites

**DOI:** 10.3390/ma12132206

**Published:** 2019-07-09

**Authors:** Wei Huang, Shicheng Wei, Yujiang Wang, Bo Wang, Yi Liang, Yuwei Huang, Binshi Xu

**Affiliations:** National Key Laboratory for Remanufacturing, Academy of Army Armored Forces, Beijing 100072, China

**Keywords:** FeCO_3_/RGO, solvothermal method, formation mechanism, microwave absorption properties

## Abstract

A novel composite of FeCO_3_ nanoparticles, which are wrapped with reduced graphene oxide (RGO), is fabricated using a facile one-spot solvothermal method. The composite consists of a substrate of RGO and FeCO_3_ nanoparticles that are embedded in the RGO layers. The experimental results for the FeCO_3_/RGO composite reveal a minimum refection loss (−44.5 dB) at 11.9 GHz when the thickness reaches 2.4 mm. The effective bandwidth is 7.9 GHz between 10.1 and 18 GHz when the refection loss was below −10 dB. Compared to GO and RGO, this type of composite shows better microwave absorption thanks to improved impedance matching. Overall, this thin and lightweight FeCO_3_/RGO composite is a promising candidate for absorbers that require both strong and broad absorption.

## 1. Introduction

Because of the ubiquity of electronic devices, electromagnetic radiation, and, in particular, signal interference have become a global problem [1,2,3]. As a result, big efforts have been made to reduce electromagnetic pollution and other related problems. One promising approach is the use of high-performance microwave absorbing materials (MAMs). Graphene, a relatively new carbon-based material, has excellent properties such as high electron mobility, high permittivity and a high specific surface area, which can dampen electromagnetic waves effectively using polarization relaxation [4,5,6,7]. However, pure graphene can reflect most of the electromagnetic waves, resulting in being unsuitable for MAM due to their poor impedance matching.

Fortunately, it is possible through to combine graphene with magnetic materials to overcome this problem [8,9]. Most of the related studies are focused on soft magnetic materials, which have high magnetic loss due to natural resonance, and they can produce better results as compounded with graphene. For example, Cui et al. [10] prepared a hollow Fe_3_O_4_@RGO composite by a facile route. The minimum reflection loss is −41.89 dB at 6.7 GHz. In the range of 1–4 mm, the reflection loss of nanocomposite thickness is less than −10 dB at 3.4 GHz to 13.6 GHz. Wang et al. [11] loaded MnFe_2_O_4_ nanoparticles on RGO sheets by one-step hydrothermal method. The minimum reflection loss of MnFe_2_O_4_/RGO is −32.8 dB at 8.2 GHz with the thickness of 3.5 mm, and the absorption bandwidth with the reflection loss below-10 dB is up to 4.8 GHz (from 7.2 to 12 GHz). Feng et al. [12] synthesized ZnFe_2_O_4_@SiO_2_@RGO core-shell microspheres by “coating-coating” method. The minimum reflection loss of the sample with a thickness of 2.8 mm can reach −43.9 dB at 13.9 GHz. In recent years, the composite of paramagnetic FeCO_3_ and RGO has shown great brilliance in the field of batteries due to its excellent electrochemical properties [13,14,15]. However, as far as we know, the microwave absorption properties of FeCO_3_/RGO, especially its magnetic loss characteristic spectrum, have not been investigated.

Therefore, FeCO_3_/RGO composites were synthesized using a one-pot solvothermal method. To reveal the microwave absorption mechanism of the FeCO_3_/RGO composite, the frequency dependence of both complex permittivity and the reflection-loss formation were studied and compared with GO and RGO. The outcome of this study can aid the development of light-weight and broadband electromagnetic-wave absorbers.

## 2. Experimental

10.8 g FeCl_3_·6H_2_O, 7.2 g urea, 5 g PVP and 1.2 g nano-iron powder were added into a 400 mL graphene-oxide slurry (purchased from Qitaihe Baotailong New Materials Co., Ltd. (Qitaihe, China) Containing GO 3.3 mg/mL). The mixture was dispersed, aided by ultrasonic treatment for 30 min, to form a homogeneous solution, and subsequently put into a 500 mL Teflon-lined stainless-steel autoclave, where it was kept at 200 °C for 12 h. After cooling to room temperature, the reaction products were washed with deionized water and alcohol, three times. Finally, the reaction products were dried in a vacuum furnace at 60 °C for 24 h. 

The morphology, structure, surface elements, and the electromagnetic parameters were analyzed using SEM, TEM, XRD, XPS, and VNA, field emission scanning electron microscopy (FE-SEM, Nava Nano FE-SEM450/650, Eindhoven, Netherlands), transmission electron microscopy (TEM, LI-BRA200, Oberkochen, German), X-ray diffraction (XRD, D/MAX-2500PC, Rigaku, Tokyo, Japan), X-ray photoelectron spectroscopy (XPS, PHI5300, Ulvac-Phi, Tokyo, Japan), and Vector network analyzer (VNA, PNA-N5244A, AGILENT, Santa Clara, CA, USA),respectively. The electromagnetic parameters of the measured samples were prepared by mixing the products (60%) with molten paraffin wax (40%) and placing them into a toroidal mold (*Φ_in_* = 3 mm, *Φ_out_* = 7 mm) with a thickness of 2.0–3.0 mm. The test software (AGILENT, Santa Clara, CA, USA) is 85071 and the calibration part is 85050D. Before the test, the permittivity of air was measured as an evaluation of the calibration effect. 

## 3. Results and Discussion

**Figure 1:**
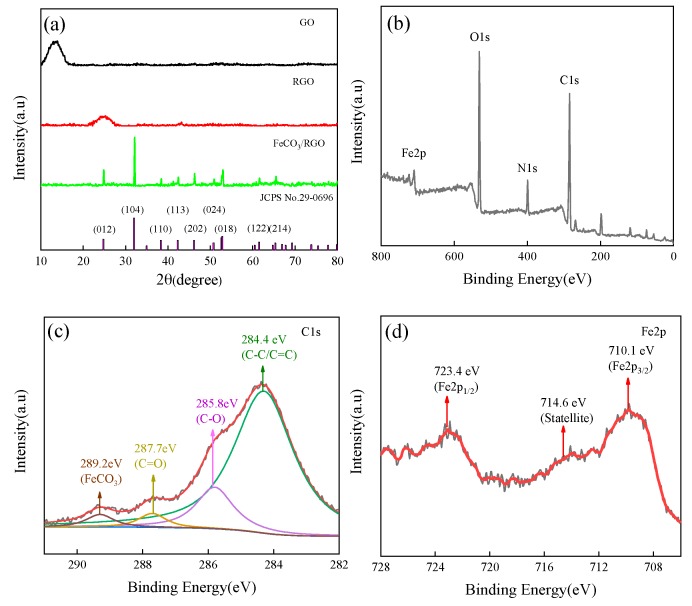
(**a**) XRD patterns for graphene oxide, (GO), reduced graphene (RGO), and FeCO_3_/RGO; XPS spectra of FeCO_3_/RGO; (**b**) wide scan; (**c**) C1s spectrum; (**d**) Fe2p spectrum.

**Figure 2:**
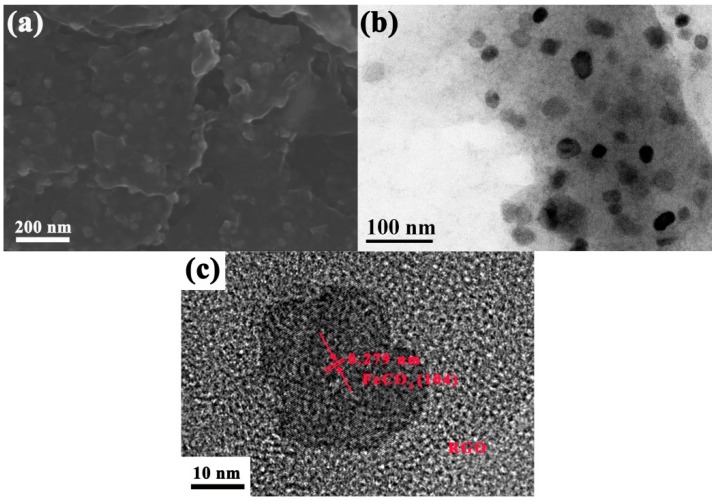
(**a**) SEM; (**b**) TEM; (**c**) HRTEM images of FeCO_3_/reduced graphene oxide (RGO).

Figure 1a shows the XRD patterns of GO, RGO, and the FeCO_3_/RGO composite. There is a broad peak at 13.4° in GO (pattern a), which corresponds to the (001) reflection of GO [16]. The broad peak at 25.2° and the disappearance of the peak at 13.4° (pattern b) indicate that GO was reduced to RGO. The XRD patterns of the FeCO_3_/RGO composite (pattern c) show that all the diffraction peaks match JCPS No.29-0696, which confirms that the FeCO_3_/RGO composite was indeed obtained. The disappearance of the RGO peaks in FeCO_3_/RGO [17] due to the uniform distribution of FeCO_3_ particles between graphene layers (Figure 2), which prevents the interlayer aggregation of RGO sheets, causes the diffraction intensity, i.e., the RGO peak, to be much smaller than for FeCO_3_.

The XPS survey spectrum of FeCO_3_/RGO (Figure 1b) shows that the composite consists of Fe, O, C, and N. Four peaks were detected (284.4 eV, 285.8 eV, 287.7 eV, 289.2 eV) in C1s spectrum (Figure 1c), which correspond to C=C/C–C, C–O, C=O, and FeCO_3_ [13], respectively. As shown in Figure 1d (Fe2p), two peaks appear at 710.1 eV and 723.4 eV, which correspond to Fe2p_3/2_ and Fe2p_1/2_. Furthermore, a satellite peak of Fe2p_3/2_ appears at 714.1 eV [18]. These characteristic peaks confirm the presence of FeCO_3_/RGO. The formation of FeCO_3_ can be derived from the following chemical equations:

(1) $$CO{\left(NH_{2}\right)}_{2}+3H_{2}O\overset{}{\to }2NH_{4}{}^{+}+CO_{2}+2OH^{-}$$

(2) $$CO_{2}+2OH^{-}\overset{}{\to }CO_{3}{}^{2-}+H_{2}O$$

(3) $$2Fe^{3+}+Fe\overset{}{\to }3Fe^{2+}$$

(4) $$Fe^{2+}+CO_{3}{}^{2-}\overset{}{\to }FeCO_{3}$$

Figure 2 shows the SEM, TEM, and HRTEM images of FeCO_3_/RGO. Polyhedron-like FeCO_3_ nanoparticles with a diameter of 20~40 nm were evenly embedded in layers of lamellar RGO. The formation of FeCO_3_ can be also proved by Figure 2c, and the space between two lattice fringes is 0.279 nm, corresponding to (104) plane of FeCO_3_. During the reduction process, the uniform distributions of nanoparticles in the RGO layers can prevent GO from agglomerating and the formation of a FeCO_3_-RGO conductive network, which might help facilitate dielectric loss.

**Figure 3:**
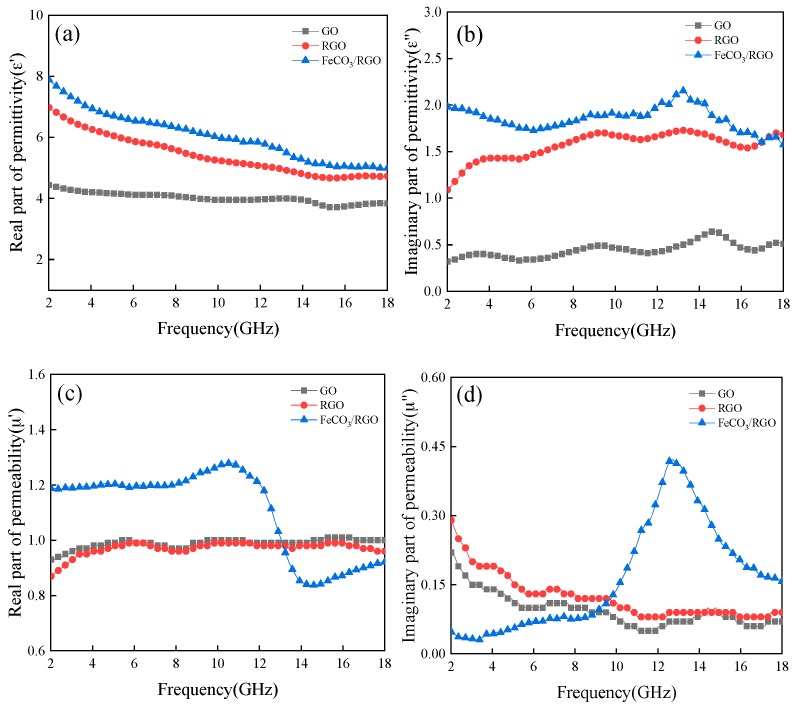
(**a**) *ε*′; (**b**) *ε*″; (**c**) *μ*′; (**d**) *μ*″ for graphene oxide (GO), reduced graphene oxide (RGO), and FeCO_3_/RGO.

Figure 3 shows the frequency-dependent electromagnetic properties of GO, RGO, and FeCO_3_/RGO between 2 and 18 GHz. Figure 3a,b illustrate the associated real (*ε*′) and imaginary (*ε*″) complex permittivity. The values of *ε*′ show the same trend, *ε*′ decreases with increasing frequency. Furthermore, the *ε*′ of FeCO_3_/RGO is higher than for both GO and RGO due to the enhanced polarization characteristics. Also, the *ε*″ of FeCO_3_/RGO is larger than for both GO and RGO due to higher conductivity [19]. Figure 3c,d depict the real (*μ*′) and imaginary (*μ*″) complex permeability of the composites. The complex permeability of GO and RGO varies similarly with frequency, indicating that there is little effect of the reduction reaction on the magnetic properties of GO. However, the observed trend for FeCO_3_/RGO is different from GO and RGO. The complex permeability of FeCO_3_/RGO varies greatly between 10 GHz and 16 GHz because the magnetic FeCO_3_ nanoparticles can produce natural resonance loss and exchange resonance loss (due to size effect, surface effect, and spin wave excitation) [20,21,22,23].

As well known, reflection loss (*R*_L_) can assess and characterize microwave absorption performance. According to the transmission line model, *R*_L_ of a metal-backed microwave absorption layer can be calculated by the following formulas [24]:

(5) $$R_{L}=20\mathrm{lg}\left|\frac{Z_{\mathrm{in}}-Z_{0}}{Z_{\mathrm{in}}+Z_{0}}\right|$$

(6) $$Z_{\mathrm{in}}=\sqrt{\frac{\mu_{r}}{\varepsilon_{r}}}\tanh[j\frac{2\pi df}{c}\sqrt{\mu_{r}\varepsilon_{r}}]$$

**Figure 4:**
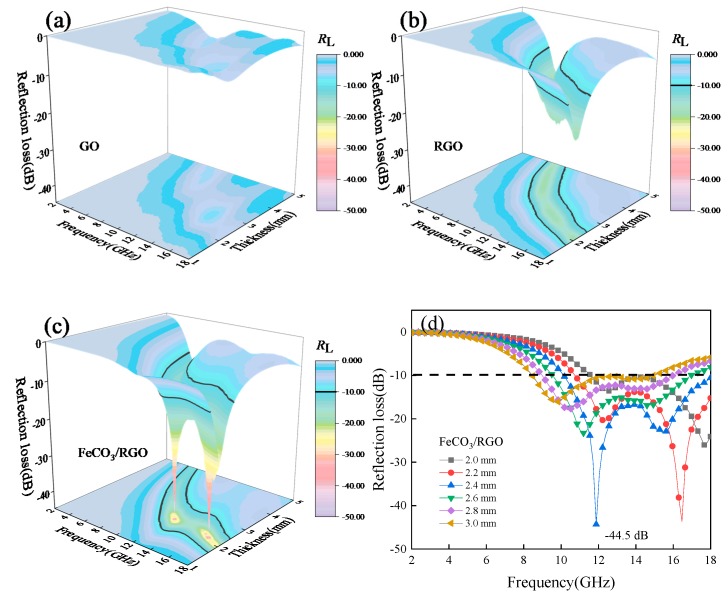
3D *R*_L_ plots of (**a**) graphene oxide (GO); (**b**) reduced graphene oxide (RGO) and (**c**) FeCO_3_/RGO; (**d**) *R*_L_ curves of FeCO_3_/RGO with 2~3 mm.

Here, *Z*_in_ is the input impedance of the absorber, *Z*_0_ is the impedance of free space (*Z*_0_ is generally 1), *ε*_r_ and *μ*_r_ are the complex permittivity and permeability, *c* is the speed of light in vacuum, *d* is the thickness of the absorber, and *f* is the microwave frequency. 3D theoretical *R*_L_ plots of the composites are shown in Figure 4a–c. It can be observed that with the reduction of GO and the introduction of FeCO_3_, the microwave absorption properties improves significantly. FeCO_3_/RGO nanocomposites show excellent microwave absorption properties as the thickness between 2 mm and 3 mm. *R*_L_ curves of composites versus frequency is shown in Figure 4d. *R*_L(min)_ appears in X and Ku band as thickness in the range of 2–3 mm and shifts to lower frequency with increasing thickness. When the thickness is 2.4 mm, FeCO_3_/RGO shows optimal microwave absorption and reaches a *R*_L(min)_ of −44.5 dB, while the corresponding bandwidth is less than −10 dB is 7.9 GHz (10.1~18 GHz). It is noteworthy that the effective bandwidth of FeCO_3_/RGO can reach up to 6 GHz and keep steadily when the thickness is 2~3 mm. Table 1 lists some reported microwave absorption composites of soft magnetic based material, graphene-based material, and FeCO_3_/RGO composite prepared in this work. Notably, FeCO_3_/RGO composite not only displays a promising negative *R*_L_ value, but also has a wide effective absorption bandwidth due to the good impedance matching.

## 4. Conclusions

The FeCO_3_/RGO composite produced and investigated in this study is a novel material with excellent microwave absorption. The composite can not only effectively facilitate electromagnetic loss but also improve impedance matching. Specifically, the refection loss at 11.9 GHz, when the composite thickness is 2.4 mm, reaching a minimum of −44.5 dB, and the effective bandwidth is 7.9 GHz (from 10.1 to 18 GHz). In addition, we observed very stable broad characteristics for a thickness range of 2–3 mm. Because of the good properties mentioned above, this composite is can be regarded as an excellent microwave absorber with the potential for many commercial applications.

## Figures and Tables

**Table 1:** Microwave absorption performances of the soft magnetic material-based, graphene material-based composite compared with FeCO_3_/reduced graphene oxide (RGO).

Sample	RL (dB)	Effective Bandwidth (GHz) (RL < −10 dB)	Thickness (mm)	Wt. (%)	Reference
Fe_3_O_4_/RGO	−41.89	4.2	2.5	50	[10]
Fe_3_O_4_@SnO_2_/RGO	−45.5	3	4.5	50	[25]
MnFe_2_O_4_/RGO	−29	4.88	3	10	[1]
NiFe_2_O_4_/RGO	−58	4.08	2.7	27	[26]
RGO/MWCNTs/ZnFe_2_O_4_	−23.8	2.6	1.5	50	[27]
RGO/MWCNTs/CoFe_2_O_4_	−46.8	3.4	1.6	50	[28]
RGO/Cu_2_O/Cu	−51.8	4.1	1.3	50	[29]
CoS_2_/RGO	−56.9	4.1	2.2	50	[30]
FeCO_3_/RGO	−44.5	7.9	2.4	60	This work
